# An Incidental Diagnosis of Extraosseous Ewing’s Sarcoma in the Kidney

**DOI:** 10.7759/cureus.53916

**Published:** 2024-02-09

**Authors:** Saleh Al-Gburi, Vinutha Thonse, Omer Abdalla, Manal Kumar

**Affiliations:** 1 Urology, Arrowe Park Hospital, Wirral University Teaching Hospital NHS Foundation Trust, Wirral, GBR; 2 Urology, Mosul Medical College, University of Mosul, Mosul, IRQ; 3 Pathology, Arrowe Park Hospital, Wirral University Teaching Hospital NHS Foundation Trust, Wirral, GBR

**Keywords:** urology surgery, urology and oncology, ewing sarcoma (es), kidney, robot-assisted partial nephrectomy

## Abstract

Ewing's sarcoma is generally observed in the skeletal and connective tissues of paediatric individuals. The occurrence of extraosseous neuroectodermal tumours is uncommon. Renal Ewing’s sarcoma usually presents with flank pain, haematuria, or as an abdominal mass. Immunohistochemistry and fluorescence in situ hybridization (FISH) techniques are essential in its diagnosis and differentiation from other tumours.

We present asymptomatic renal Ewing’s sarcoma in a 19-year-old female patient who was diagnosed incidentally, and the CT scan confirmed a 2.8 cm left mid-pole renal mass suggestive of malignancy. She was managed with a robotic partial nephrectomy. Tumour immunohistochemistry and the FISH technique confirmed the diagnosis of Ewing’s sarcoma. The patient made an uneventful recovery and was referred for chemotherapy.

This case report illustrates that despite the aggressiveness of the tumour, it can be detected earlier despite an asymptomatic presentation and be successfully treated with nephron-sparing surgery and chemotherapy.

## Introduction

Ewing's sarcoma (ES), alternatively referred to as primitive neuroectodermal tumours (PNET), encompasses a collection of undifferentiated neoplasms that arise from the neuroectodermal tissue. It is generally observed in the skeletal and connective tissues of paediatric individuals [1]. The occurrence of extraosseous primitive neuroectodermal tumours (PNETs) affecting the genitourinary system, specifically the kidney, bladder, epididymis, and prostate, is exceptionally uncommon [2].

Primary renal Ewing's sarcoma exhibits a highly aggressive course characterised by rapid growth and early metastasis to the lung, bone, and lymph nodes, which ultimately leads to an unfavourable prognosis [3, 4].

We describe a rare case of renal Ewing’s sarcoma in a 19-year-old girl that was found incidentally during an ultrasound investigation, which was then treated with a nephron-sparing approach. To the best of our knowledge, this is the first asymptomatic case diagnosed incidentally in the literature, and the third case treated with nephron-sparing surgery successfully.

## Case presentation

A 19-year-old female patient with severe learning difficulties was referred to the urology department for an ultrasound investigation due to deranged liver function tests. The ultrasound of the abdomen detected a well-defined echobright lesion associated with internal vascularity in the lower pole of the left kidney. Her medical background was significant for severe learning difficulties and seizures in early childhood with unknown aetiology. She has no history of epileptic episodes and is not taking antiepileptic medications. She is currently on iron supplements and has no allergies. She had a history of a corneal transplant for keratoconus and there was no clinical suspicion of syndrome. There was no family history of renal cancer. 

**Figure 1:**
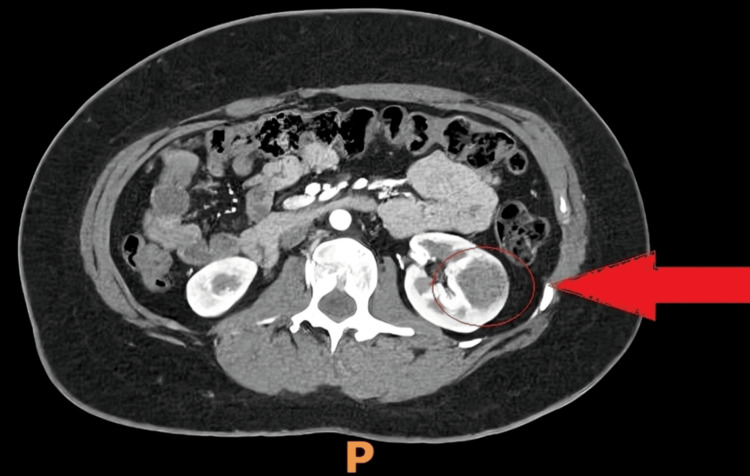
An axial view of CT thorax and abdomen with contrast showing a 2.8 cm left mid-pole renal lesion (red arrow and circle).

**Figure 2:**
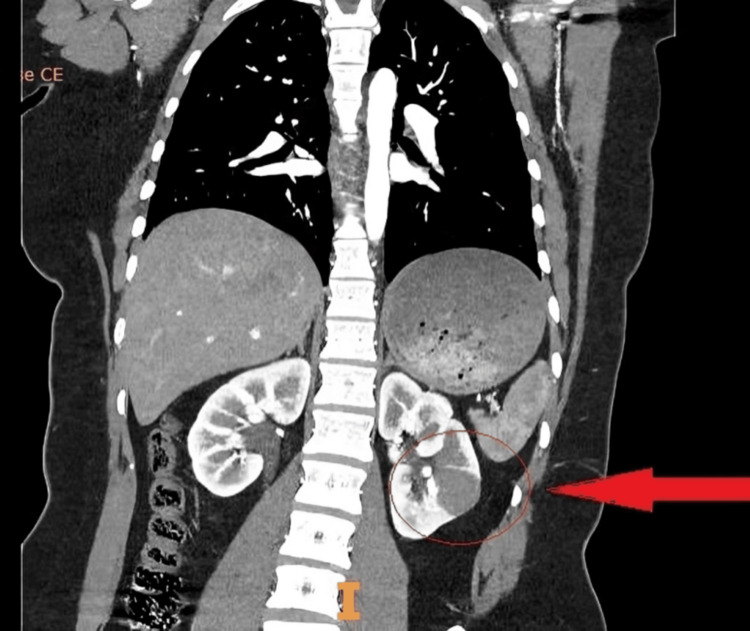
A Coronal view of CT thorax and abdomen with contrast showing a 2.8 cm left mid-pole renal lesion (red arrow and circle).

She had a CT scan, which showed a 2.8 cm left mid-pole renal mass suggestive of malignancy. The CT thorax and abdomen with contrast showed no evidence of metastasis. The CT findings are illustrated in Figures 1-2. 

**Figure 3:**
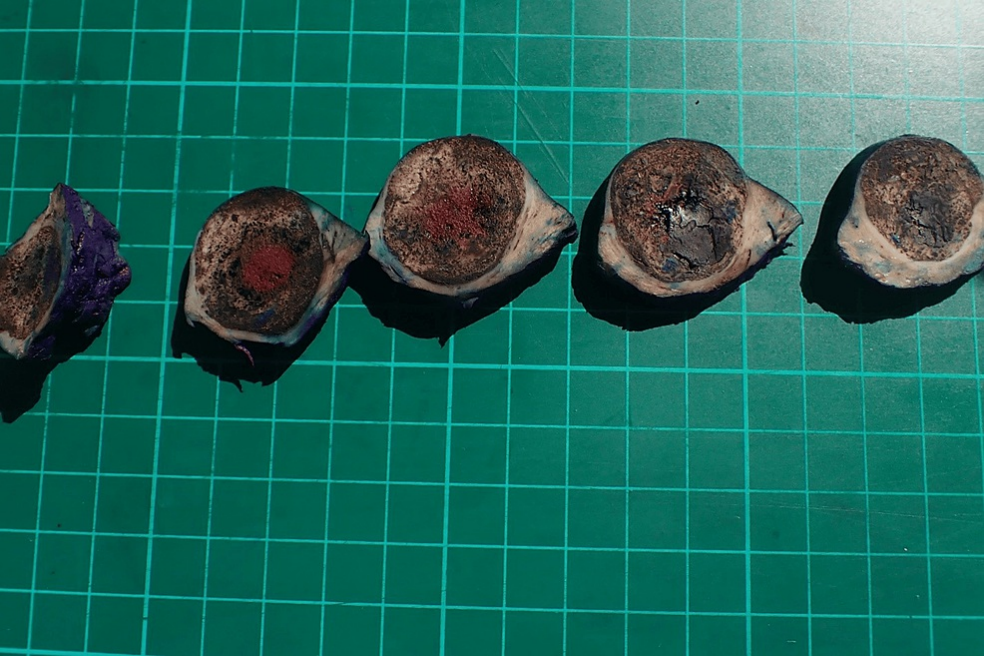
Macroscopic specimens of the kidney stone

**Figure 4:**
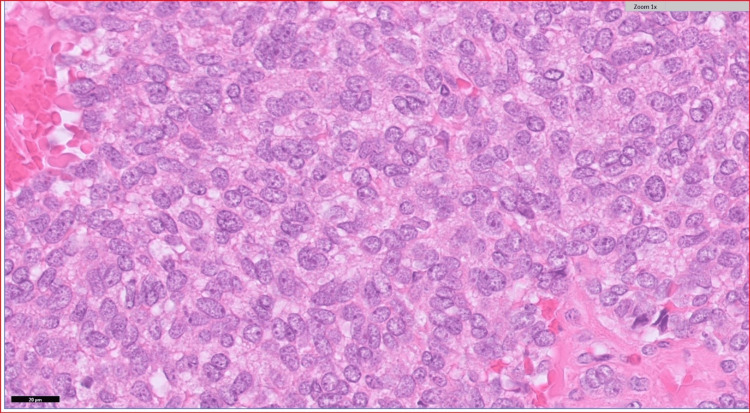
Microscopic features of the kidney tumour

The patient underwent an uneventful left robotic-assisted laparoscopic partial nephrectomy. The histology demonstrated a solid tumour measuring 35 x 33 x 20 mm. The cut surface was circumscribed and variegated, 1 mm from the capsular and 2 mm from the parenchymal resection margin. Microscopically, the tumour was composed of cells arranged in nests. These cells are round to ovoid with fine chromatin, a slightly prominent nuclear outline, a few prominent nucleoli, and increased abnormal mitotic figures. There was a slight overlapping of nuclei. In some places, the cells take on a spindle-like appearance. Prominent fibrous septae are seen dividing this tumour into nodules. No obvious clear cell area or necrosis is seen. Its macroscopic and microscopic features are illustrated in Figures 3-4. 

**Figure 5:**
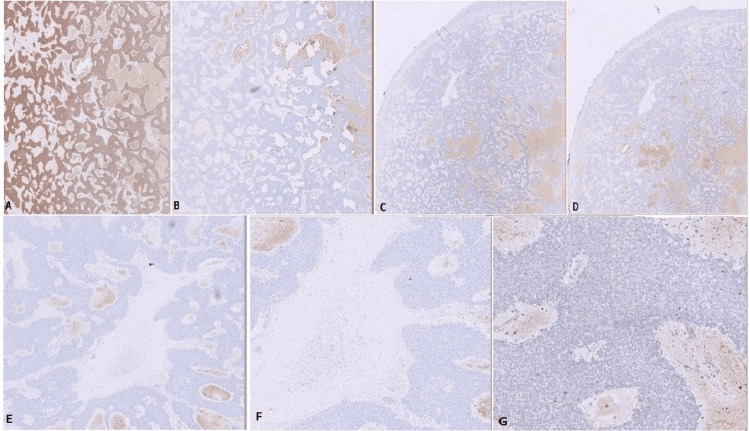
Immunohistochemistry (A) S100: positive; (B) AE1/AE3: negative; (C) SOX-10: negative; (D) CD56: negative; (E) Chromogranin: negative; (F) Synaptophysin: negative; (G) Ki-67: negative.

**Figure 6:**
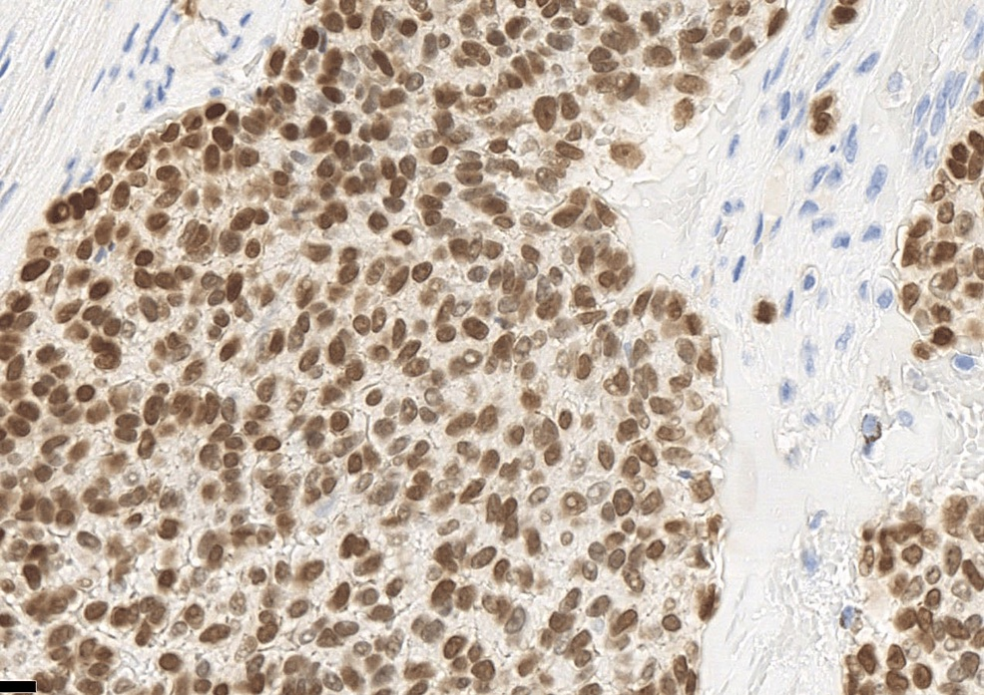
Immunohistochemistry showed that the tumour positive for NKX2.2.

**Figure 7:**
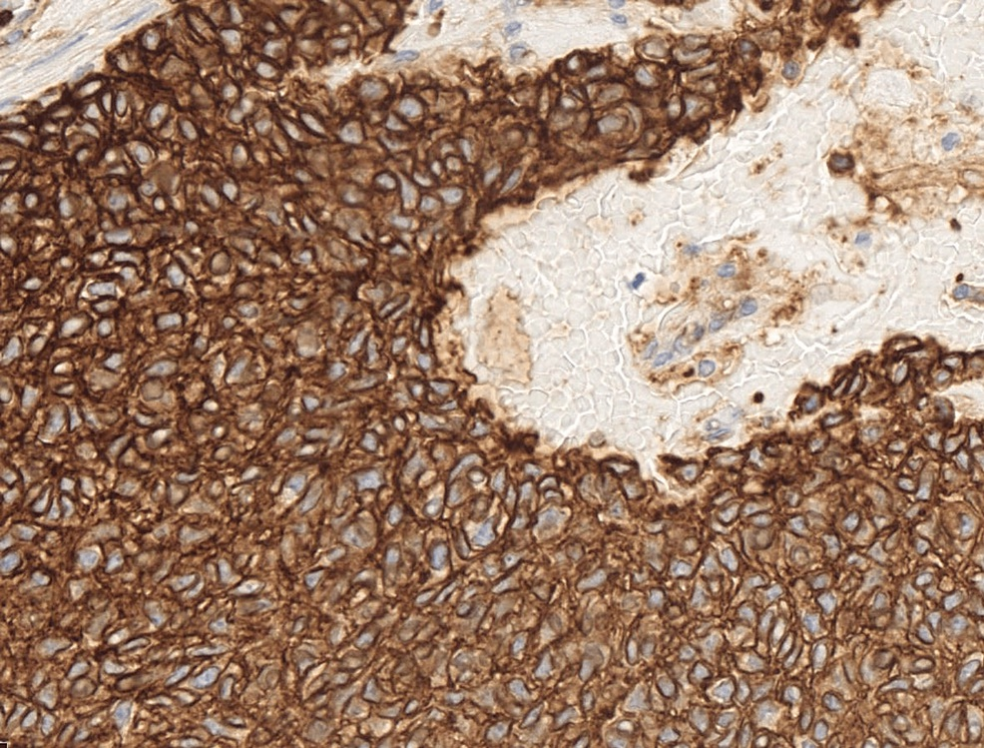
Immunohistochemistry showed that the tumour positive for CD 99.

On immunohistochemistry, the tumour was positive for vimentin and S100. The tumour was negative for CD56, chromogranin, synaptophysin, EMA, AE1/AE3, CD34, Bcl-2, Melan A, SOX-10, inhibin, and CD57 (Figure 5). On additional immunohistochemistry, tumour cells showed strong membranous staining for CD99 and nuclear positivity for NKX2. 2. Flourescent in situ hybridization showed EWSR1::FLI1 rearrangement, confirming the diagnosis of Ewing’s sarcoma. The immunohistochemistry is illustrated in Figures 5-7. 

The patient was discharged after two days of admission and was referred for chemotherapy after a discussion with the multidisciplinary uro-oncology team (MDT). The oncology team decided to start adjuvant chemotherapy, which included alternative weeks of vincristine 2mg, doxorubicin 40mg, cyclophosphamide 2000 mg in the first week, and ifosfamide 5040 mg with etoposide 260 mg in the next week over 14 cycles of treatment. She developed neutropenia and neuropathy that required changing the doses of chemotherapy. She finished the 14 cycles of chemotherapy and follow-up CT scans of the thorax, abdomen, and pelvis with contrast done three and six months after the operation were normal and showed no recurrence or distant metastasis. Adjuvant radiotherapy was not indicated for the patient as she had a negative resection margin. 

**Figure 8:**
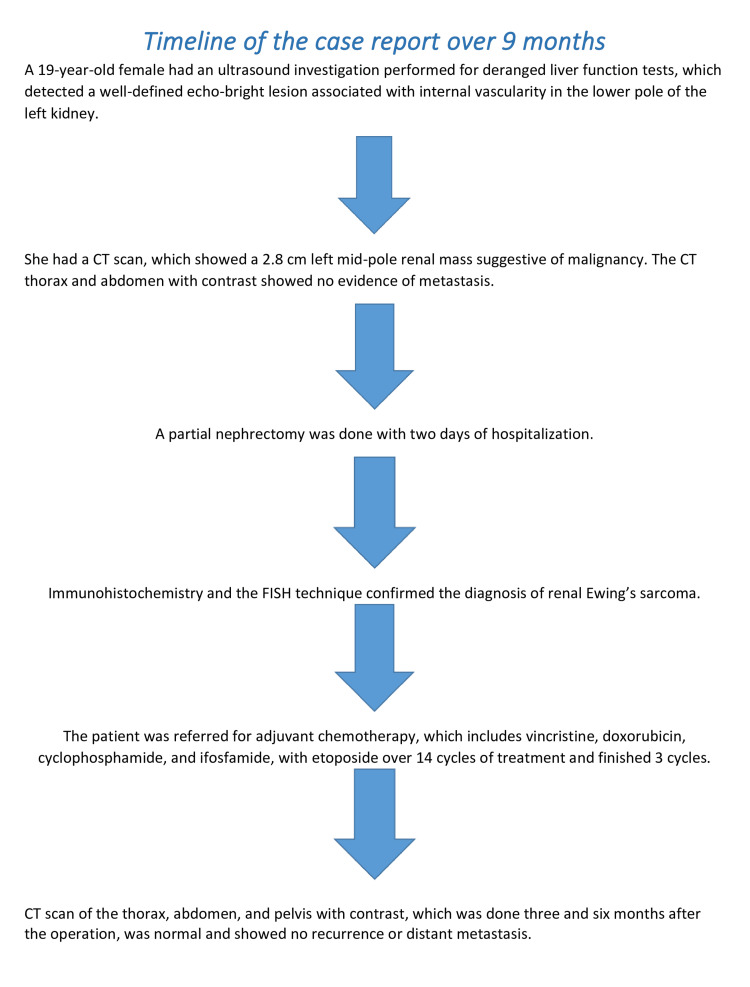
Timeline of the case report over nine months FISH: fluorescence in situ hybridization

## Discussion

The first documented case of Ewing’s sarcoma in the kidney was reported in 1975 [5]. The clinical manifestations of these sarcomas are generally nonspecific, with patients commonly reporting pain and the presence of palpable lumps [6, 7]. Due to its innately aggressive characteristics, patients commonly manifest with severe disease and early metastases [8]. In the literature, the reported cases of Ewing’s sarcoma were commonly presented with haematuria, however, this case presents the first reported asymptomatic incidental case.

The utilisation of immunohistochemistry and the fluorescence in situ hybridization (FISH) technique were crucial for differentiating Ewing's sarcoma from other tumours of the same histological features, such as Wilms tumour, neuroblastoma, rhabdomyosarcoma, and lymphoma. Ewing's sarcoma is distinguished by a stable chromosomal translocation, namely t (11:22), which occurs between the EWS gene located on chromosome 22q12 and the FLI-1 gene located on chromosome 11q24, which was identified using the FISH technique [6, 9].

Given the remarkable rarity of Ewing's sarcoma in the kidney, the established approach for managing this tumour remains insufficient and primarily relies on therapeutic protocols used for osseous Ewing's sarcoma. Successful treatment requires a multimodal approach that consists of surgery, chemotherapy, and radiotherapy [6]. The chemotherapy regimen commonly employed in the treatment of Ewing's sarcoma consists of a combination of drugs, vincristine, doxorubicin, and cyclophosphamide, which are administered in alternate cycles with ifosfamide and etoposide [7]. Adjuvant radiation may be warranted in positive resection margins or recurrences. Most of the cases in the literature underwent radical nephrectomy, however, in our case, the patient underwent successful nephron-sparing surgery (NSS). Suzuki et al. and Hakky et al. have reported successful treatment with nephron-sparing surgery for renal Ewing’s sarcoma cases with no recurrence at one year; however, those cases had presented with abdominal pain and flank pain, respectively, while our case was asymptomatic [10, 11].

Despite the use of intensive therapeutic interventions, the overall prognosis for individuals diagnosed with metastatic disease remains poor [6, 7, 12, 13].

Renal Ewing's tumour exhibits a higher level of aggressiveness compared to its occurrence in other locations. It exhibits a high tendency for local recurrence and early metastasis to lymph nodes, lungs, liver, and bone [14].

## Conclusions

Ewing's sarcoma can affects the kidneys and typically manifests with symptoms of flank pain, hematuria, or the presence of an abdominal mass. It is usually more aggressive compare to Ewing’s sarcoma in other sites. The diagnosis of Ewing's sarcoma typically occurs via surgical intervention or biopsy, utilising immunohistochemistry and FISH techniques, and it is generally a highly aggressive tumour, prone to early metastasis.

Despite the disease's aggressive nature, effective treatment of asymptomatic Ewing's renal sarcoma is possible if detected in its early stages. Therefore, it can be effectively managed by nephron-sparing surgery and chemotherapy.
